# Condylar alteration in three subtypes of temporomandibular disorder based on U-HRCT: a cross-sectional study

**DOI:** 10.1186/s12903-025-07557-z

**Published:** 2025-12-23

**Authors:** Ruowei Tang, Wen Chen, Ning Xu, Xiaoqing Yuan, Xuan Bai, Bihe Zhang, Xidong Zhang, Zhenghan Yang, Xiaofeng Huang, Zhenchang Wang, Pengfei Zhao, Ning Zhang

**Affiliations:** 1 Department of Radiology, Beijing Friendship Hospital, Capital Medical University, 95 Yong’an Road, Xicheng District, Beijing, 100050 China; 2 Department of Stomatology, Beijing Friendship Hospital, Capital Medical University, Beijing, 100050 China; 3 Hebei Key Laboratory of Clinical Pharmacy, The Fourth Hospital of Hebei Medical University, Shijiazhuang, Hebei 050011 China; 4 MRI Room, Imaging Center, The First Affiliated Hospital of Xi’an Jiaotong University, Yulin Hospital, Yulin, 719000 China; 5 Department of Oral and Maxillofacial Plastic and Traumatic Surgery, Beijing Stomatological Hospital, Capital Medical University, Beijing, 100070 China

**Keywords:** Bone remodeling, Mandibular condyle, Osteoarthritis, Temporomandibular joint disorders, Tomography, X-Ray computed

## Abstract

**Background:**

To investigate osseous remodeling pattern of the condyle across different subtypes of temporomandibular disorder (TMD) using 3D model generated from ultra-high-resolution CT (U-HRCT).

**Methods:**

This single-center, retrospective study enrolled patients with unilateral TMD who underwent both U-HRCT and magnetic resonance imaging from November 2021 to June 2024. Temporomandibular joints (TMJs) were categorized into three TMD subtypes: disc displacement with reduction (DDWR), disc displacement without reduction (DDWoR), and osteoarthritis (OA). Condylar morphology parameters, including length, width, height, total volume, anterior volume, and posterior volume, were measured on 3D model derived from U-HRCT images by two independent observers. Affected and unaffected TMJs of the same patient were compared using paired sample *t*-test, and inter-group differences among TMD subtypes were assessed using two-way ANOVA with Bonferroni correction.

**Results:**

A total of 115 participants (mean age 34.6 ± 13.4 years, 103 females) were included. Compared with unaffected side, affected TMJ showed significantly smaller height (DDWR, DDWoR), width (OA), and volumetric data (all groups). Inter-group comparison of affected TMJ revealed significant differences in condylar height and total volume between DDWR and OA (*p* = 0.007 and 0.001, respectively). The posterior volume was the lowest in OA (399.71 ± 123.43 mm^3^), followed by DDWoR (517.51 ± 179.30 mm^3^) and DDWR (542.11 ± 153.76 mm^3^), while the anterior volume was smallest in DDWoR (309.49 ± 107.27 mm^3^), followed by OA (336.92 ± 128.81 mm^3^) and DDWR (361.50 ± 110.03 mm^3^).

**Conclusion:**

U-HRCT-based 3D models reveal condylar osseous remodeling patterns across TMD subtypes, and posterior volume may serve as a potential osseous remodeling indicator to differentiate TMD subtypes.

**Supplementary Information:**

The online version contains supplementary material available at 10.1186/s12903-025-07557-z.

## Introduction

Temporomandibular disorder (TMD) is prevalent in general population across all age groups, with reported prevalence exceeding 31% and 11% in adults/elderly and children/adolescent, respectively [1]. While a small proportion of patients remain asymptomatic, most patients afflicted with TMD experience clinical symptoms such as joint pain, clicking, limited mouth opening, and noise [2, 3]. Diagnosis of TMD is established on a combination of clinical history, oral, and imaging examination [3]. Based on Diagnostic Criteria for Temporomandibular Disorders (DC/TMD), disc displacement with reduction (DDWR) and disc displacement without reduction (DDWoR) are classified as biomechanical disorders, whereas osteoarthritis (OA) represents a form of degenerative joint disease characterized by osseous changes, and these conditions were regarded as three most common subtypes of TMD [4].

Osseous alterations in the temporomandibular joint (TMJ), including absorption, erosion, sclerosis, and osteophyte, indicate bony repair and regeneration and are collectively referred to as osseous remodeling [5, 6]. Osseous remodeling is a dynamic, load-dependent process that occurs when joint loading exceeds the adaptive capacity of the articulating surface [7]. Specifically, ongoing condyle remodeling has been recognized as a key mechanism underlying functional impairment and symptom development in TMD [8].

Although osseous remodeling of the condyle has been widely studied, most research has focused on patients with OA [9] or on post-operative changes [10, 11]. However, adaptive remodeling may begin at the early stage of TMD, potentially before radiographic abnormalities become evident [12–14]. Existing studies on condylar morphology have reported inconsistent results. For example, one study proposes that condylar volume is significantly affected by bony erosion [15], whereas another investigation reveals no correlation between condylar volume and specific bony changes, such as erosion or osteophyte formation [16]. These inconsistencies highlight the need to investigate condylar morphology across TMD subtypes (from DDWR to OA) to elucidate the underlying pathological mechanism of TMD and to develop a more precise treatment plan.

Computed tomography (CT), renowned for its excellent visualization of bony structure, is recommended for assessing osseous condition of TMJ [6, 17]. Hence, the aim of the current study was to identify osseous changes of the condyle across three subtypes of TMD through 3D model generated from ultra-high-resolution CT (U-HRCT).

## Materials and methods

This retrospective, cross-sectional study, approved by the local bioethics committee (approval no. 2022-P2-366-01 and 2024-P2-061-02) and in accordance with the Helsinki Declaration, was conducted in a tertiary referral center from November 2021 to June 2024. The informed consent from the participants was obtained.

### Eligible participants

The inclusion criteria were based on clinical examination and disc position as follows for patients [3]: (1) clinically diagnosed TMD with symptoms such as joint pain, clicking, limited mouth opening, and noise, and (2) completion of both ultra-high-resolution CT (U-HRCT) and MR examinations. The exclusion criteria were patients with: (1) bilateral TMD for the purpose of direct comparison (*n* = 688), (2) no positive imaging findings from U-HRCT or MR images (*n* = 128), (3) incomplete clinical data (*n* = 78), (4) history of oral and maxillofacial surgery (*n* = 12), (5) prior joint injury (*n* = 13), and (6) severe motion artifacts interfering with image interpretation (*n* = 28). Finally, 115 patients with 230 TMJs were enrolled.

**Fig. 1:**
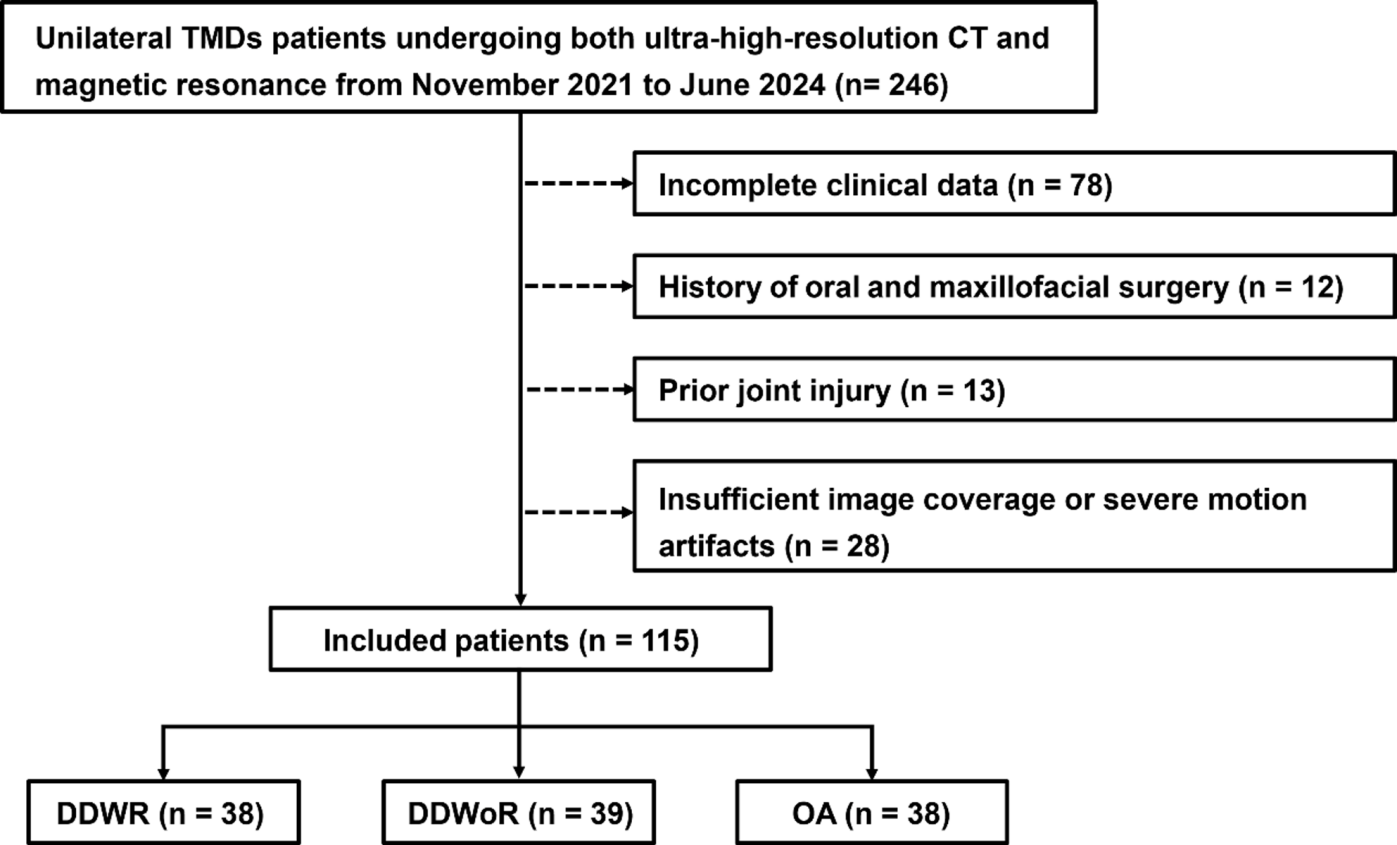
Flowchart of the study

The included patients were categorized into three groups based on imaging criteria: (1) DDWR, defined as an anteriorly displaced disc (posterior band of the disc located anterior to the 11–12 o’clock position relative to the condylar head) on closed-mouth proton density-weighted sagittal MR image that returned to a normal position on the open-mouth image; (2) DDWoR, defined as an anteriorly displaced disc on both closed- and open-mouth MR images; and (3) OA, defined by presence of any of the following appearance on CT image: osteophytic formation, bony erosion, subcortical cyst, flattening, subcortical sclerosis, or condyle deformity. Finally, a total of 38, 39, and 38 patients were assigned to the DDWR, DDWoR, and OA groups, respectively (Fig. 1). The contralateral, unaffected TMJ of each patient served as the normal control for pair-wise comparison. Demographic data (age and sex), clinical manifestation (onset duration and chief complaint), and treatment strategy (observation, splint therapy, pharmacological, laser, sclerotherapy treatment, and surgical treatment) were collected for each patient.

### U-HRCT protocols

All participants underwent U-HRCT examination for the bilateral TMJs using a U-HRCT scanner (Ultra3D, LargeV) at 100–110 kVp and 120–180 mAs with a field-of-view of 65 mm. Both slice thickness and interval were set to 0.1 mm. Isotropic axial images were obtained, and the exposure dose ranged from 82.99 to 98.11 µGy [18].

### Image analysis

#### Image processing

All CT datasets were anonymized by removing patient identifier and replacing it with a random study ID. Images were imported into the Mimics software (version 16.0, Materialise N.V.) for data conversion, 3D segmentation, and visualization of TMJ. The generated 3D images were compared with the original images via multiplanar reformation and 3D volume-rendering to ensure consistency. Compared to measurement on 2D image, 3D image can provide more accurate result regarding measurement of anatomical structures [19, 20]. Therefore, all subsequent measurement of condylar morphology was performed using the generated 3D model. Two board-certified observers (R.T. and W.C. with 9 and 8 years of experience, respectively), blinded to clinical history, physical-examination, and MR finding, performed measurement independently. After an interval of four weeks, one of the observers (R.T.) performed a second measurement.

### Standard observation plane

**Fig. 2:**
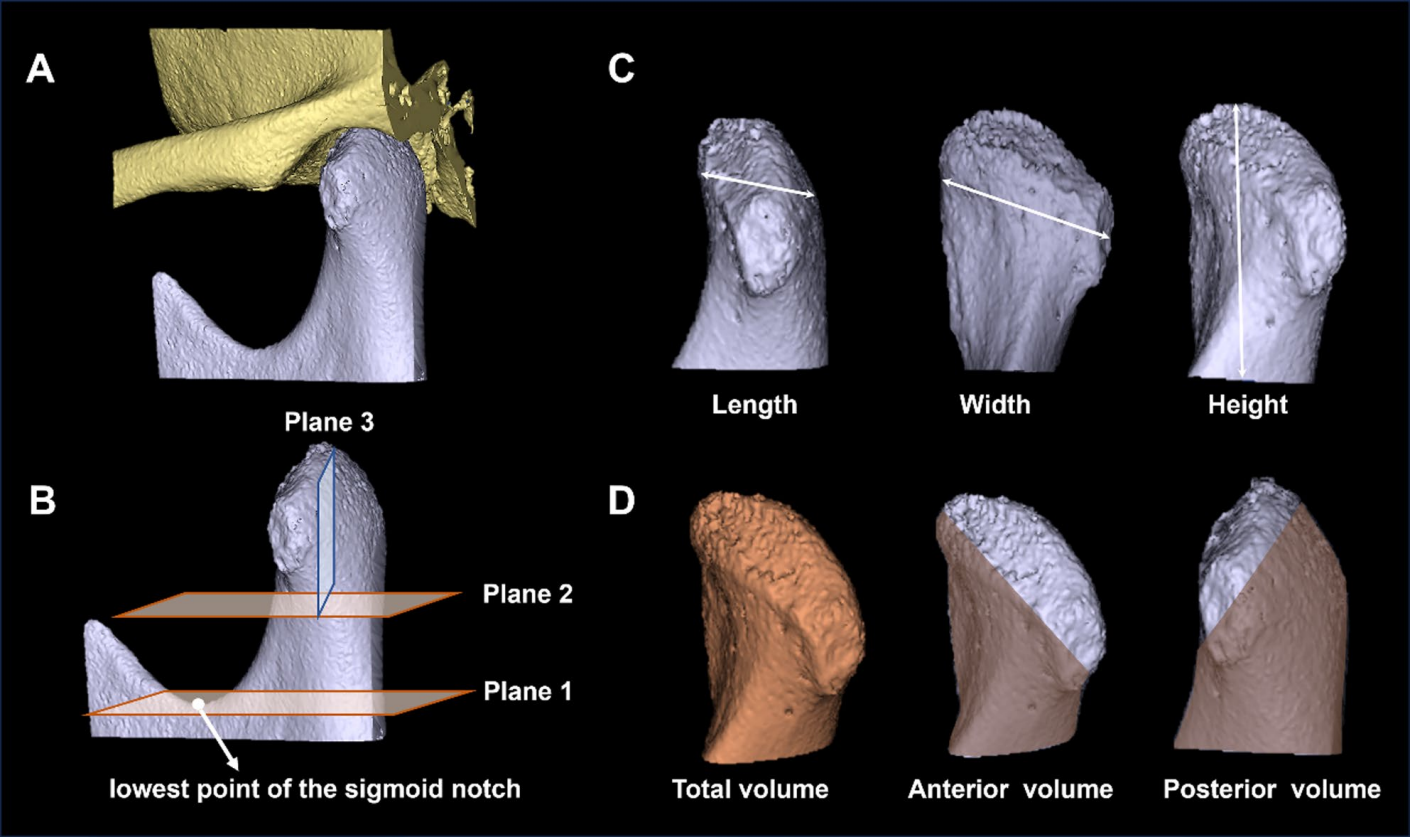
Measurement of condylar morphology on U-HRCT -based 3D models (**A**). **B** Standard observation planes. Plane 1 is the horizontal plane passing through the lowest point of the sigmoid notch, Plane 2 is positioned mid-height of the condyle, and Plane 3 is the coronal plane passing through the condylar apex. **C** measurement of condylar length, width, and height. **D** measurement of total, anterior, and posterior volumes (orange)

First, a reference plane (Plane 1) was established parallel to the Frank fort horizontal plane and passing through the lowest point of the sigmoid notch. A second horizontal plane (Plane 2) was positioned halfway between the condylar apex and Plane 1. A third plane (Plane 3), oriented in the coronal direction and perpendicular to Plane 1, was aligned to pass through the apex of the condylar (Fig. 2).

### Measurement of the condylar morphology

For precise identification of reference points, the two observers independently reviewed the 3D model and selected landmark on the condyle indepedently. The detailed measurements were as follows (Fig. 2):

1. Length: the maximal anterior-posterior distance was determined by identifying the most anterior (A) and the most posterior (P) points on the condylar surface. These points were defined as the loci with the greatest projection relative to the overall condylar contour. A linear distance between A and P was recorded as the condylar length.
2. Width: the maximal mediolateral distance was measured by identifying the most medial (M) and the most lateral (L) points on the condyle. the distance between M and L points was recorded as the condylar width.
3. Height: the perpendicular distance from the most superior point of the condyle to Plane 2 was defined as the condylar height.
4. Volume: the total condylar volume was defined as the volume enclosed between the most superior point of the condyle and Plane 2. The anterior and posterior condylar volumes were determined based on Plane 3, with anterior volume referring to the region anterior to Plane 3 and posterior volume referring to the region posterior to Plane 3.

### Statistical analysis

A sample size was calculated using PASS 15 (NCSS) based on an effect size derived from preliminary pilot data. Assuming a two-tailed significance level of 0.05 and a desired power of 0.9, the analysis indicated that a minimum of 28 subjects per group was required. Consequently, a consecutive enrollment strategy was adopted, and recruitment for each subgroup was concluded once the target sample size was reached.

The statistical analysis was conducted using SPSS 26.0 (IBM). Quantitative data were expressed as mean ± standard deviation (SD) or median (interquartile range, IQR), while qualitative data were reported as numbers (percentages). The intraclass correlation coefficient (ICC) was used to calculate the intra- and inter-observer agreement for quantitative metric as follows: poor (< 0.50), moderate (0.50–0.75), good (0.75–0.90), and excellent (> 0.90). Paired-sample *t*-test was used to assess difference between the affected and unaffected TMJs for each participant. Comparison of morphological data among the three TMD subtypes (DDWR, DDWoR and OA) was conducted using a two-way ANOVA with Bonferroni correction. To account for multiple comparisons, the adjusted threshold significance for the Bonferroni test was set at *p* < 0.017 (0.05/3). For all the other tests, a *p* value < 0.05 was considered statistically significant.

## Results

### Demographic characteristics and condyle morphology of the entire cohort

A total of 115 participants (mean age 34.6 ± 13.4 years, females 103/115, 89.6%) were enrolled, consisting of 38 patients with DDWR, 39 with DDWoR, and 38 with OA. All participants exhibited unilateral symptom, allowing the contralateral TMJ to serve as the normal control. The chief complaint of the affected TMJ included noise (62/115, 53.9%), pain (52/115, 45.2%), clicking (47/115, 40.9%), and limited mouth opening (40/115, 34.8%). Additional demographic and clinical characteristics are summarized in Table 1.

**Table 1:** Demographic and clinical characteristics of the participants

Demographic and clinical data	Values
Age, years	
Mean ± SD	34.6 ± 13.4
Median, IQR	31 (25–40)
Range	19–82
Sex, n (%)	
Male	12 (10.4)
Female	103 (89.6)
TMD subtype, n (%)	
DDWR	38 (33.0)
DDWoR	39 (33.9)
OA	38 (33.0)
Onset duration (month, IQR)	6 (1.5–24)
Chief complaint, n (%)^*^	
TMJ noise	62 (53.9)
TMJ pain	52 (45.2)
TMJ clicking	47 (40.9)
Mouth opening limitation	40 (34.8)
Treatment strategy, n (%)	
Observation and mood adjustment	43 (37.4)
Orthodontics/occlusal splint therapy	29 (25.2)
Pharmacological, laser, or sclerotherapy treatment	26 (22.6)
Surgical treatment	17 (14.8)

*SD* Standard deviation, *IQR* Interquartile range, *TMD *Temporomandibular disorder, *TMJ *Temporomandibular joint, *DDWR* Disc displacement with reduction, *DDWoR* Disc displacement without reduction, *OA* Osteoarthritis

^*^The affected TMJ may present with one, two or more clinical manifestations, thus the sum of this entry is 201 TMJs

### Intra- and inter-observer agreement

The intra-observer consistency showed good to excellent agreement (ICCs 0.85 to 0.90) in measuring condylar length, width, height, and volumes, while inter-observer consistency ranged from moderate to good (ICCs ranged from 0.75 to 0.83). The average values from the two observers were used for comparison among DDWR, DDWoR, and OA.

### Pair-wise comparison between the affected and unaffected TMJs

For the entire 230 TMJs, condylar dimensions were as follows: length 8.02 ± 1.49 mm, width 18.45 ± 2.44 mm, and height 9.83 ± 1.61 mm. The volumetric data were 898.96 ± 254.96 mm^3^ for the total volume, 373.91 ± 126.21 mm^3^ for the anterior volume, and 522.83 ± 179.26 mm^3^ for the posterior volume.

**Fig. 3:**
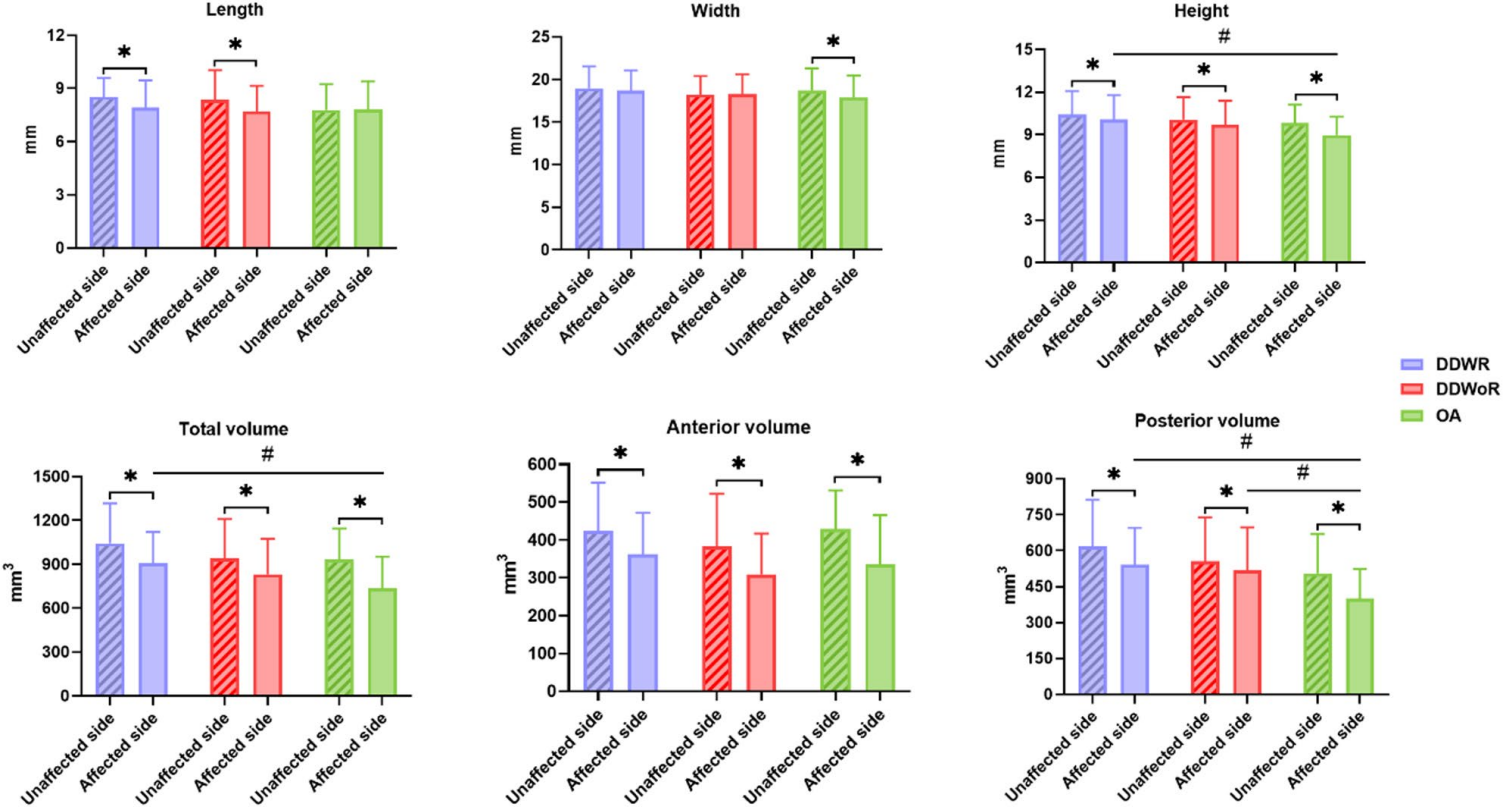
Pair-wise and inter-group differences in condylar morphology among DDWR, DDWoR, and OA. Pair-wise differences between affected and unaffected TMJs within each subtype(*), and inter-group differences among subtypes(#). DDWR = purple, DDWoR = red, OA = green

**Fig. 4:**
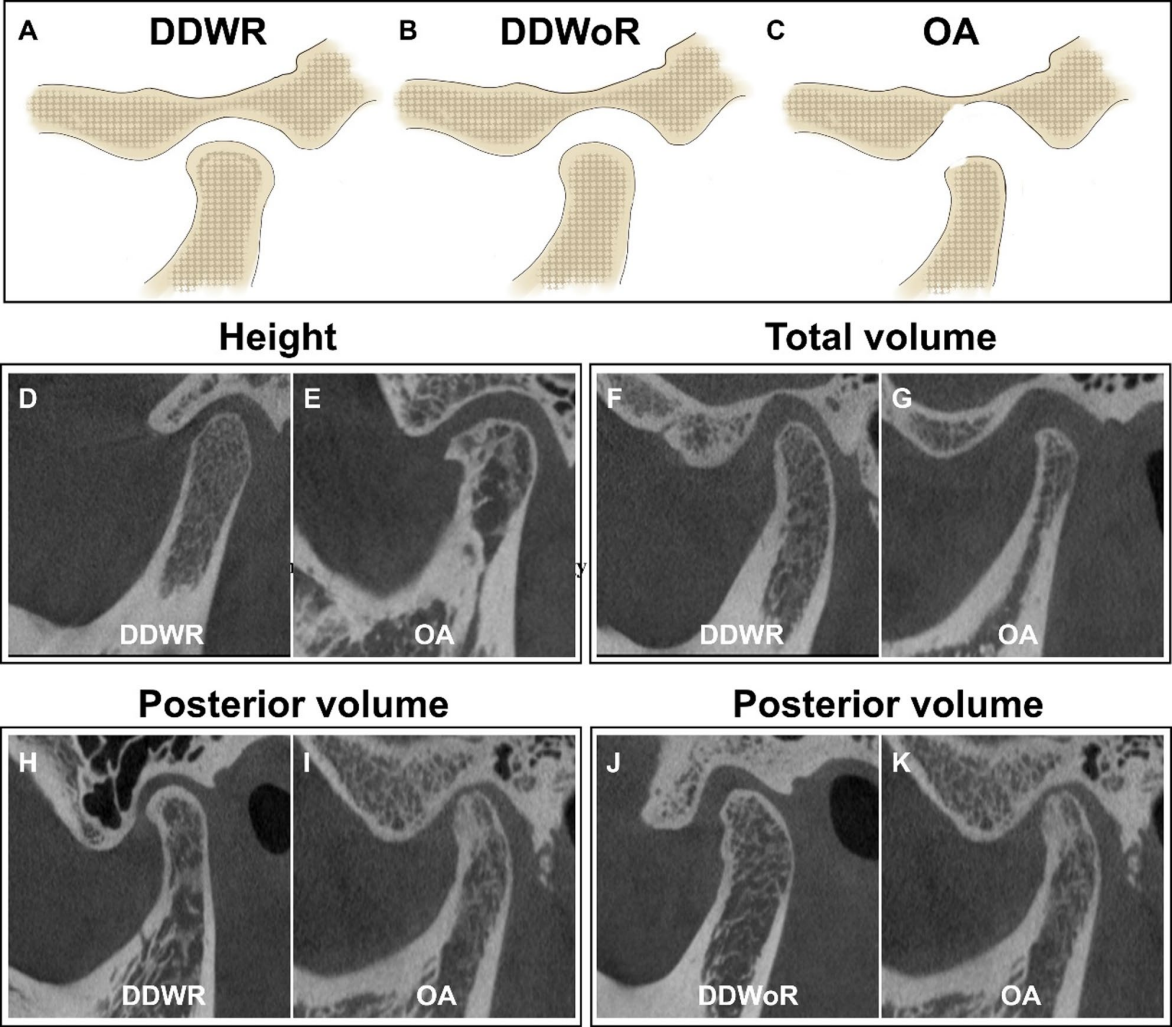
Inter-group difference in condylar morphology among DDWR, DDWoR, and OA. **A**-**C** Schematic diagram of condylar osseous remodeling. **D**-**E** Inter-group comparison of condylar height with significant difference. **F**-**G** Inter-group comparison of condylar volume. **H**-**I** Posterior volume difference between DDWR and OA. **J**-**K** Posterior volume difference between DDWoR and OA

Pair-wise comparison revealed that unaffected TMJ had greater condylar length than affected side in both the DDWR (8.53 ± 1.06 mm vs. 7.91 ± 1.55 mm, *p* = 0.006) and DDWoR (8.36 ± 1.67 mm vs. 7.70 ± 1.45 mm, *p* = 0.023) groups, but not in the OA group. Conversely, significant differences in condylar width were only found between the unaffected and affected sides in the OA group (18.73 ± 2.57 mm vs. 17.89 ± 2.58 mm, *p* = 0.005), while no significant differences were observed in the DDWR or DDWoR groups. Additionally, condylar height was smaller in the affected TMJ compared to the unaffected side in all three groups (Table 2, Figs. 3 and 4).

**Table 2:** Pair-wise comparison of condylar morphology between the affected and unaffected TMJs in the TMD subtypes

Condylar morphology	DDWR (*n* = 38)			DDWoR (*n* = 39)			OA (*n* = 38)		
	Affected side	Unaffected side	*p* value	Affected side	Unaffected side	*p* value	Affected side	Unaffected side	*p* value
Length (mm)	7.91 ± 1.55	8.53 ± 1.06	0.006	7.70 ± 1.45	8.36 ± 1.67	0.023	7.81 ± 1.59	7.79 ± 1.46	0.950
Width (mm)	18.66 ± 2.41	18.98 ± 2.56	0.314	18.27 ± 2.32	18.22 ± 2.17	0.861	17.89 ± 2.58	18.73 ± 2.57	0.005
Height (mm)	10.06 ± 1.72	10.41 ± 1.68	0.034	9.69 ± 1.70	10.05 ± 1.60	0.025	8.96 ± 1.31	9.82 ± 1.29	< 0.001
Volume (mm^3^)									
total volume	905.45 ± 214.93	1043.61 ± 273.11	< 0.001	831.28 ± 243.65	940.77 ± 269.47	< 0.001	738.42 ± 215.21	934.89 ± 208.98	< 0.001
anterior volume	361.50 ± 110.03	424.47 ± 126.83	0.005	309.49 ± 107.27	383.21 ± 138.96	< 0.001	336.92 ± 128.81	429.34 ± 101.68	< 0.001
posterior volume	542.11 ± 153.76	617.08 ± 195.58	< 0.001	517.51 ± 179.30	555.72 ± 183.46	0.024	399.71 ± 123.43	504.11 ± 166.06	< 0.001

*TMJ* Temporomandibular joint, *TMD* Temporomandibular disorder, *DDWR* Disc displacement with reduction, *DDWoR*  Disc displacement without reduction, *OA*  Osteoarthritis

For the volumetric data, total volume was significant smaller in the affected TMJ compared than the unaffected side for all three groups (all *P*s < 0.001). Similarly, affected TMJ showed smaller anterior and posterior volumes in the DDWR (anterior 361.50 ± 110.03 mm^3^, *p* = 0.005, posterior 542.11 ± 153.76 mm^3^, *p* < 0.001), DDWoR (anterior 309.49 ± 107.27 mm^3^, *p* < 0.001; posterior 517.51 ± 179.30 mm^3^, *p* = 0.024), and OA (anterior 336.92 ± 128.81 mm^3^, *p* < 0.001; posterior 399.71 ± 123.43 mm^3^, *p* < 0.001) groups compared with the unaffected side (Table 2; Figs. 3 and 4). The average volume differences were 138 mm^3^ (10.88%), 113 mm^3^ (11.13%), and 193 mm^3^ (20.09%) for the total volume; 63 mm^3^ (9.73%), 76 mm^3^ (15.11%), and 90 mm^3^ (20.06%) for the anterior volume; and 75 mm^3^ (7.96%), 40 mm^3^ (5.85%), and 103 mm^3^ (17.65%) for the posterior volume in the affected side of DDWR, DDWoR, and OA groups, respectively.

### Inter-group difference of condylar morphology

No significant differences were found among the unaffected TMJ across all three groups (*p* > 0.017, Supplemental Table 1). After adjusting for age and sex, inter-group comparison of affected TMJ showed that DDWR group had significantly greater condylar height and total volume than the OA group (*p* = 0.007 and 0.001, respectively). In addition, posterior condylar volume was significant larger in both DDWR and DDWoR compared with OA (*p* < 0.001 and *p* = 0.001, respectively), while no significant difference was observed between the DDWR and DDWoR groups (Table 3; Figs. 3 and 4).

**Table 3:** Comparisons of condylar morphology of affected TMJ across TMD subtypes, after adjusting for age and sex

Condylar morphology	ANOVA		*p* value for Bonferroni correction^*^		
	F value	*p* value	DDWR vs. DDWoR	DDWR vs. OA	DDWoR vs. OA
Length (mm)	0.393	0.676	-		
Width (mm)	1.370	0.259	-		
Height (mm)	4.645	0.012	0.245	0.007	0.044
Volume (mm^3^)					
total volume	6.172	0.003	0.087	0.001	0.057
anterior volume	2.060	0.132	-		
posterior volume	10.130	< 0.001	0.314	< 0.001	0.001

*TMJ* Temporomandibular joint, *TMD* Temporomandibular disorder, *DDWR* Disc displacement with reduction, *DDWoR* Disc displacement without reduction, *OA* Osteoarthritis

^*^Significant difference is set as *p* value 0.05/3 = 0.017 for the Bonferroni correction

## Discussion

The current study revealed the osseous remodeling pattern in TMJ with or without TMD and compared across three TMD subtypes (DDWR, DDWoR and OA) using U-HRCT. This study confirmed that bony alteration was present in DDWR, presenting as smaller condylar length, height, and volume on the affected side compared to the unaffected side. Significant differences in condylar height and total volume were observed between DDWR and OA, and posterior volume was the only morphological parameter that distinguished DDWoR from OA. These findings underscore the dynamic nature of osseous remodeling in TMD, offering valuable insights for refining diagnostic criteria.

Unlike many other anatomical structure, TMJ exhibits a unique capacity for lifelong osseous remodeling. This is attributed to the presence of a periosteum on condylar articular surface, which contains a proliferative zone with intrinsic growth potential influenced by the dynamic forces loading on the joint [21]. In addition to that, the fibrocartilage disc distributes stress across the bony surface on TMJ [22]. As a result, remodeling can occur not only after trauma but also in situations involving articular disc displacement or excessive loading. Previous studies have defined osseous remodeling from a range of aspects, including morphology of the condyle, disc, and articular fossa, as well as their position and spatial relationship [23]. This study aimed to identify specifically the morphological alteration of the condyle across TMD subtypes, in accordance with DC/TMD classification [2, 3].

Osseous remodeling in TMD is influenced by multiple factors, such as signaling pathway, occlusion, parafunctional habits, and position of the articular disc [15, 16, 24, 25]. Studies on disc repositioning surgery have demonstrated that the position of the articular disc significantly influences the site of bony remodeling. Specifically, relocating the disc from the anterior to the posterior slope of the condyle induces new bone formation at the corresponding location [25]. These results are partially consistent with our study, which revealed that both the anterior and posterior volumes undergo remodeling in TMD, with disc displacement likely contributing to these changes. Understanding how remodeling pattern varies across TMD subtypes may help physicians tailor treatment plan to the patient’s pathological status, avoid unnecessary invasive procedure, and intervene decisively when needed.

Classification of TMD varies depending on the purpose of study. The most common classification is the DC/TMD [3]. In this study, we included patients with DDWR, DDWoR, and OA at a ratio of 1:1:1 to enable comparison among the three subtypes. Although comparison between DDWR and DDWoR have been frequently reported [26], fewer studies include OA, despite evidence given that up to 40% of affected TMJ potentially develop OA [27]. In this study, we have included OA to present a comprehensive view. The observation between the affected and unaffected sides in DDWR already exhibiting measurable morphological difference indicates the importance of early identification and appropriate management, including addressing detrimental habit, stabilizing occlusion, and considering orthodontic or occlusal splint therapy. In addition, the ability of U-HRCT detecting subtle osseous remodeling change in TMD supports its applicability for early detection and accurate diagnosis.

In OA, a smaller posterior condylar volume emerged as a notable imaging feature, indicating posterior condyle may be the most adaptive region. This finding contrasts with the conventional view that the anterior region, as the primary functional stress-loading zone, is most adaptive. Specifically, we found that the posterior volume was consistently lower across the three groups, whereas the anterior volume was smaller in DDWoR (vs. DDWR) and larger in OA (vs. DDWoR). The relatively larger anterior portion in the OA group may reflect the presence of osteophyte formation, while the smaller anterior portion in DDWoR group may relate to bony resorption. Nevertheless, the total volume remained smaller in DDWoR and OA, suggesting that changes in the anterior portion do not compensate for the reduced volume in the posterior portion, and that the posterior component contributes more substantially to the volumetric difference observed in OA. Although correlations between condylar dimension and TMD is well-documented, discrepancies exist among previous studies. Some report that the smaller condylar length, width, and height may be associated with TMD [28, 29]. Our study confirmed that condylar height effectively distinguished affected TMJ from unaffected ones, but it showed limited ability to differentiate between DDWR and DDWoR, or between DDWoR and OA, with a notable difference only between the DDWR and OA groups. The varying results may be attributed to factors such as sample size, disease spectrum, and measurement method [16]. In contrast to previous studies, our study observed a greater condylar length in the OA group, which may be related to new bone formation.

In this study, U-HRCT was utilized to visualize the bony architecture of TMJ. This newly-developed imaging device was designed to image the temporal bone region. Although MR can detect osseous changes, CT remains the gold standard for bone visualization. U-HRCT has been applied in depicting anatomical characteristics [30, 31] and diagnosing lesion in patients with ear diseases [32–34]. Its recent application has demonstrated superior image quality and diagnostic performance for TMD compared to commonly used cone-beam CT [35]. In this study, U-HRCT’s diagnostic value in TMD expanded to support 3D quantitative assessment, which is considered more accurate than 2D measurement [19, 20]. The improved image resolution facilitated precise semiautomatic segmentation and reconstruction of the condyle, ensuring accuracy of subsequent measurement.

This study has several limitations. First, its cross-sectional design allows comparison across diagnostic subtypes but does not support evaluation of temporal changes, and unmeasured factors, such as symptom duration, etiology, and prior treatment, may have influenced inter-group comparison. Second, although age and sex were adjusted, other clinical variables including pain severity, parafunctional habits, occlusal and dental-arch morphology, and psychosocial or muscular factors were not included; these should be incorporated in future multi-factor, prospective studies. Third, only patients with unilateral TMD were included to enable within-subject comparison, improving internal validity but potentially limiting generalizability; future studies should include bilateral cases. Fourth, Although calculation of the sample size was performed and the included number of patients was sufficient, subgroup comparison for other covariates may require a larger cohort of patients. Therefore, larger, multi-center, multi-ethnic cohort are needed. Finally, imaging-based evaluation alone cannot fully represent the multifactorial nature of TMD, and integration of MR and additional clinical indicator is required to achieve a more comprehensive evaluation of TMD osseous alteration.

## Conclusions

In conclusion, analysis of U-HRCT-based 3D model shows that smaller condylar height and volume are present in DDWR, whereas OA has a more significant smaller posterior volume compared with anterior volume. Therefore, posterior condylar volume may demonstrate discriminatory ability among the three TMD subtypes and may serve as a useful marker for differentiating DDWR, DDWoR, and OA.

## Supplementary Information

Supplementary Material 1.

## Data Availability

The datasets used and/or analyzed during the current study are available from the corresponding author on reasonable request.

## Abbreviations

TMD: Temporomandibular disorder
DDWR: Disc displacement with reduction
DDWoR: Disc displacement without reduction
OA: Osteoarthritis
TMJ: Temporomandibular joint
U-HRCT: Ultra-high-resolution CT
SD: Standard deviation
IQR: Interquartile range
