# Supplementary material for: Condylar alteration in three subtypes of temporomandibular disorder based on U-HRCT: a cross-sectional study
Source: BMC Oral Health. 2025 Dec 23;25:1930. doi: 10.1186/s12903-025-07557-z (PMC12729815; doi:10.1186/s12903-025-07557-z)
Supplement: Supplementary file 1 — Supplementary Material 1. [file 12903_2025_7557_MOESM1_ESM.docx]

**Supplemental Table 1. Comparisons of condylar morphology of the unaffected TMJs among TMDs groups using ANOVA and Bonferroni correction**

| Condylar morphology | ANOVA | | *p* value (95% CI) for Bonferroni correction^*^ | | |
| --- | --- | --- | --- | --- | --- |
|  | *F* value | *p* value | DDWR *vs.* DDWoR | DDWR *vs.* OA | DDWoR *vs.* OA |
| Length (mm) | 2.893 | 0.060 | － | | |
| Width (mm) | 0.962 | 0.385 | － | | |
| Height (mm) | 1.410 | 0.248 | － | | |
| Volume (mm^3^) |  |  |  |  |  |
| total volume | 2.238 | 0.111 | － | | |
| anterior volume | 1.625 | 0.201 | － | | |
| posterior volume | 3.665 | 0.029 | 0.426 (-39.53－162.25) | 0.024 (11.43－214.52) | 0.649 (-49.28－152.50) |

DDWR = disc displacement with reduction, DDWoR = disc displacement without reduction, OA = osteoarthritis, CI = confidence interval

^*^ *p* value is set as 0.05/3=0.017 for the Bonferroni correction
